# Miscellanies

**Published:** 1841-01-01

**Authors:** 


### MISCELLANIES.

### Mr. Warburton's Medical Reform Bill.

We insert, from the Lancet, the principal clauses of this embryonic Bill. The objections to it are not trivial, and appear to be generally felt.

" A Bill for the Registration of Medical Practitioners, and for establishing a College of Medicine, and for enabling the Fellows of that College to practice Medicine in all or any of its Branches, and hold any Medical Appointments whatsoever, in any part whatsoever of the United Kingdom."

Passing over definitions, &c. we proceed to the Clauses, which declare as follows :—

II. The costs of administering the Act shall be paid by an annual tax of [*blank in the Bill*] on every registered or unregistered medical practitioner in the United Kingdom, according to the provisions of the bill, forming the fund of a " Medical Registry Account." Any deficiency to be specially supplied by Parliament.

III. From and after 1842, registers of all persons practising medicine (in chief) in England, Scotland, and Ireland, shall be made and kept by three persons, nominated by the Secretary of State for the Home Department, whose offices shall be respectively situated in the three capitals, having the registrars of births and deaths throughout England, certain schoolmasters in Scotland, and officers of the police in Ireland, as sub-registrars.

IV. V. These clauses describe the duties of the sub-registrars, and require the medical practitioner to supply to them a schedule of name, address, branch of medicine, and nature and dates of his qualifications; "but," says the clause, " if he do not hold a medical qualification, then whether it is as being a chemist and druggist that he practises medicine in chief," and if under the Apothecaries' Act, or a right acbuired by usage before that Act was passed; " or whether he practises medicine in chief without either holding a medical qualification or being a chemist and druggist." Each partner in a firm to do the same.

VI. VII. VIII. All this to be signed, returned before the 1st of April, 1843; or if no blank schedule to be filled up has been sent to the party by the sub-registrar, then the party must, before the 7th of April in every year, apply for one, to fill up and return, the sub-registrar being required to comply with the request speedily afterwards. The period and demand for such returns from medical practitioners are to be publicly advertised also. These returns are to include the names, addresses, and qualifications of "parties who practise medicine in chief in their capacity of chemists and druggists," as well as "parties who practise medicine in chief, and are not included in the division of persons who hold medical qualifications, or who do not practise in the capacity of chemists and druggists."

IX. The registers are to be printed and published *by the registrars* afresh on the 1st of August in every year, so far as regards those persons who hold what is termed in the Bill a "medical qualification."

X. Permission is given to the *Secretary of State* to publish or not, as he may think fit, the two divisions of persons who "practise medicine in chief in their capacity as chemists and druggists," and those who "practise medicine in chief without being included in either of the preceding divisions."

XI. Any one may reprint registers.

XII. Medical practitioners may "require" any person who has returned himself in a schedule to prove the actual existence of his alleged qualification.

XIII. Persons not holding a "medical qualification" shall not hold any medical office in any public institution, or any district, parochial or otherwise, benefit society, or in either of the public services.

XIV. XV. XVI. The registry of a person to commence with the date of his "return." Changes of residence to be notified to registrars, and announced in supplements to the registers.

XVII. After the 31st of July, in the year 184—, *it shall not be lawful for any unregistered person,* "even although he hold a medical qualification, *to act as a medical practitioner in any part of the United Kingdom,* any custom or thing contained in any statute, charter, gift, grant, or deed, or any by-law, regulation, or statute of any corporate body, to the contrary notwithstanding."

XVIII. The possession of a "medical qualification," by a person whose name shall appear in the registers or supplements, which *the registrars* are to be required by law to publish, shall render it lawful for him "to make any reasonable charge for any time he may have employed in professional attendance on any patient *in that part of the United Kingdom in which he is registered,*" and therein to sue for the same.

XIX. "Three medical councils, one for England, one for Scotland, and one for Ireland," shall be constituted, each to consist of 36 councillors, of whom, 12 shall be non-medical men "nominated and appointed" on the 1st of Oct. 1843, by the Secretary of State for the Home Department for the time being. Of these 12, there shall 3 of them annually, on Oct. 1st, vacate, at the bidding of the Secretary of State, their office, to be replaced by 3 other non-medical men, similarly nominated. The 24 other councillors shall be elected by those registered "medical practitioners in each respective country who hold a medical qualification," and shall be chosen, exclusively, from among the qualified electors themselves. The first of these elections to take place in the middle of September, 1843, and the registrar of each country to be on that occasion the scrutineer of the election. For the year 1844, the scrutineers to be chosen by voting-papers, by the electors, from amongst the electors. The 24 councillors to be elected in a similar manner. The voting-papers to be prepared by the registrars, and circulated by the sub-registrars, to be filled up by the electors, severally, and returned, sealed, to the sub-registrar, to be by him conveyed by post to the registrars in the respective capitals of each country, the votes of the electors to be kept secret by the registrars, they each reporting to the Secretary of State on whom the choice of the electors has fallen.

XX. Of these 24 elected medical councillors, 6 shall annually, on each 1st of October, vacate their seats, by decision of the said medical electors pronounced a fortnight previously; and who, from amongst their own body, shall replace those 6, the metropolitan registrars, and the previously elected scrutineers, presiding in each capital at the estimate of the votes, having on the 1st of August before, named an umpire between them. (This clause also directs that the 36 councillors shall indicate by lists, to the electors before each election, which 6 of the medical councillors they would " recommend the electors to cause to vacate," and whom " to elect, as six new councillors ;" but that the electors may either reject or comply with this " recommendation" as they may think fit.)

XXI. The three metropolitan councils each to elect its own chairman, by ballot, annually in October. At meetings of the councils, 6 to form a quorum. Decisions therein to follow the majority of votes. Minutes of their proceedings to be kept, printed and circulated amongst themselves and the other metropolitan councils, and open to a senate hereinafter mentioned, and the Secretary of State.

XXII. Of these three councils of 108 persons, 12 members are to be annually chosen from each council to form a united *Medical Senate* of 36 persons. Of each 12, four persons are to be chosen from the non-medical division.

XXIII. This " Medical Senate of the United Kingdom," is, on the 4th of October in each year, in and after 1842, to meet in London, at a place named by the Secretary of State, and choose annually a president. Its proceedings to be conducted like those of the councils.

XXIV. V. The senate may make by-laws, to be laid before Parliament, for its own regulation, and to be binding also on the aforesaid councils, " and on all fellows and matriculated students of the college hereinafter directed to be founded, and on all the examiners appointed by the said several councils, and on all the officers and servants of the senate or of the said several councils."

XXVI. VII. The councils may make regulations for giving effect to the bylaws of the medical senate ; which regulations, however, the senate may subsequently disallow.

XXVIII. The senate may attend all meetings of the councils, and take part in their discussions ; or attend the courts of the " Examiners."

XXIX. There shall be founded a " College of Medicine of the United Kingdom." Its " first fellows" shall consist of all the elected medical councillors of Oct. 1, 1843 ; and its future additional fellows shall be constituted of the like councillors for other years, all to be fellows for life ; together with such other registered persons, possessing " medical qualifications," as the senate may, by by-laws made by the senate, pronounce to be eligible for election as fellows of the said college.

XXX. This senate shall, on commencing its duties, make by-laws to define what " medical qualification," possessed by a medical practitioner, shall entitle him to claim to be a fellow of the said college, without subjecting him to an examination in medicine. The before-mentioned councils shall " make regulations for carrying such by-law into effect ;" and when any person so qualified shall apply for admission to the fellowship, the said council shall ballot for him, and reject or admit him according as the majority of their votes may decide.

XXXI. Persons not already in medical practice may be admitted to the fellowship upon examination, as follows :—The said senate shall make by-laws to define " the *examinations*, to which all persons claiming to be entered in the books of either of the councils, as *matriculated students of the said college*, shall previously to their being so entered, be subjected," and the *age* they shall have attained, and " touching the *course of instruction* to be pursued by students *subsequent* to their matriculation, and touching the medical *institutions* and *schools*, corporate or unincorporated, in the United Kingdom or in foreign parts, which shall be deemed *competent* for *the instruction of students in medicine ;* and touching the *registration* of matriculated students during their course of instruction ; and touching the *age* which persons admitted to examination for the fellowship of the said college shall be required to have attained ;" and touching the *examiners* whom the several councils shall have appointed, and touching the times and modes, and subjects of medical examination. And the senate may " relax the rigour of such by-laws in favour of those students whose course of medical instruction may be advanced towards completion when those by-laws first come into operation." The *councils* are to *appoint the examiners,* who are to "examine persons claiming to be entered in the books of the council as matriculated students of the said college." And also appoint the examiners of candidates for the fellowship of the said college. The examiners are to report to the council what candidates they pass, and then such candidates shall be entered or admitted as matriculated students or fellows, as the case may be.

XXXII. The senate may empower each of the council "to ballot for the *expulsion from the college* of any fellow thereof domiciled in the country to which such council belongs, who may have been tried for and found guilty of committing any infamous crime or offence."

XXXIII. Any fellow of the college may, at his own request, be examined by direction of the senate, as a candidate for a certificate from the council, certifyfying his proficiency in medicine, or surgery, or midwifery, or pharmacy, or some other special branch of practice.

XXXIV. No councillor can be an examiner.

XXXV. It shall "be lawful for every fellow of the said college to practise as a surgeon-apothecary, or general practitioner of medicine, in any part whatsoever, of the British dominions;" or in the same capacity to any hospital, gaol, union, society, or other public place or body; and to "compound and dispense any medicine he may prescribe for his own patients," any where; and to sue for charges for medicine, operations, or attendance; and to receive any number of pupils or apprentices; and every such fellow shall be entitled "to all exemptions from serving on juries, inquests, &c. and all other exemptions to which surgeons or apothecaries are already entitled."

XXXVI. Fellows of the said college "who shall have received from any such council a certificate" of his proficiency in medicine, and "who shall also be a graduate in medicine in any university in the United Kingdom," shall, in addition to his other before-mentioned privileges as a fellow of the said college, "be also entitled to practise as a physician in any part of the British dominions, and to act as a physician to any hospital," &c. "or, after undergoing such examination as any duly constituted medical board may deem requisite, may serve as a physician in the navy or army, &c. and be entitled to every already existing privilege of a physician."

XXXVII. This clause makes a similar declaration with regard to *surgeons,* who are fellows of the said college, giving them, on obtaining an examination, a certificate of proficiency in surgery from one of the said councils, and already possessing a surgical qualification from some other college, or faculty, or university, entitling them to "act as surgeons in any part of the British dominions."

XXXVIII. The same as regards a fellow of the said college who may have received from one of the councils a certification that he has, on examination, "been found to be proficient in the art and business of an *apothecary*;" if, also, he be "a member, or licentiate, or certified proficient of some society of apothecaries of the United Kingdom, or of the faculty of Physicians and Surgeons of Glasgow, or of the Royal College of Surgeons of Edinburgh." Such apothecary may act as an apothecary anywhere in any part of the British dominions, &c.

XXXIX. "Fellows of the college who have received a certificate of proficiency in *midwifery,* may act as *surgeon apothecaries,* or, if they be graduates in physic, as *physicians to any lying-in hospital* in any part of the British dominions." (*Marginal note.*)

XL. Fellows of the college who have received a certificate of their knowledge of the treatment of *lunatics,* may act as surgeon-apothecaries, or, if they be graduates in physic, as physicians to any lunatic hospital or asylum in the British dominions.

XLI. The senate may, if they think proper, under by-laws, purposely made by them, exempt any candidate for the fellowship of the college from the examinations, or from any part of the examinations, which such candidate would be liable to undergo before admission, if that candidate have already acquired "a medical qualification" in the United Kingdom, or a degree in medicine in some university abroad, of which the said senate may approve. The council to which he may apply shall then ballot for the admission of the said candidate.

XLII. Chemists and druggists may voluntarily apply to be examined by examiners named by the said senate. Their examination, "shall relate to the Latin language, the interpretation of prescriptions, the pharmacopœia, the articles of the materia medica, the quantities of different simple or compound medicines which may safely be administered to patients, chemistry, practical and pharmaceutical, and botany." To every person passing this examination the council shall grant a certificate of proficiency therein; and if such person desire a registrar to register him in a certain annual list of certified chemists and druggists, he shall be so registered. And "any person so certificated shall be entitled to carry on the business of a chemist and druggist in any part of the British dominions." And "if any person so certificated shall carry on the business of a chemist and druggist in any town, the population of which" amounts to —— inhabitants, then "the laboratory or shop of such person shall be approved of as a *school for pharmacy* by each of the said medical councils."

XLIII. Every student "shall be deemed to have completed a proper course of instruction in *pharmacy,*" who shall have attended such laboratory or shop, as above-mentioned, or any apothecary's or hospital, or medical practitioner's shop or laboratory, *recognized* by the medical council of the country, during a continuous period of not less than —— years or months; a longer or shorter period for either kind of shop or laboratory as the by-laws of the said council may demand.

XLIV. The said senate may prepare and publish a national *pharmacopœia* for the use of all medical men.

XLV. All *medical assistants* in England, Scotland, and Ireland, to be *registered* from January 1, 1845, by means of a form, to be called "The Medical Assistant's Notice and Schedule," recording their name, age, address, medical qualification (if any), and those of the medical practitioners to whom they may be assistants. And when the annual registers of the medical practitioners of the country are published, those registers shall respectively contain, in juxta position with their names, those of the medical assistants whom they may severally be at that time employing.

XLVI. All *medical students* in the United Kingdom, who intend to obtain, by and by, a "medical qualification," are to be *registered* by the parties with whom they may be apprentices or pupils,—dates, ages, family domiciles, the hospitals, or medical schools they may be attending in that twelve months; the specific courses of lectures and demonstrations they may be attending: the name of every professor, lecturer, demonstrator, and the date and duration of their attendances on the instructions of those persons; and all professors and teachers in every university, college, school, hospital, or dispensary, in the United Kingdom where medicine is taught, are to assist by the registrations of their pupils in rendering this registration of pupils and their studies complete.

XLVII. No part of any course of medical instruction, excepting that for which a student shall have been *registered,* shall be allowed by the senate to qualify the student for *admission to examination.*

XLVIII. If any medical student be *studying* "*in foreign parts,*" with the intention of becoming a candidate for examination as aforesaid, "no part of any such foreign course of instruction shall" qualify him to be admitted to such examination, unless he *once a quarter* sends notice thereof, with full particulars (certified by the foreign teachers), to one of the registrars at home.

### MEDICAL REFORM.

WE have inserted two Bills, which will shortly be brought before the Legislature. Most persons seem to think them impracticable. Mr. Wakley promises another. The difficulty is, that we do not start de novo. There are Colleges in esse, with funds, interests, influences, and an actual state of medical society created by them, to contend with. Yet Reform is fermenting in the minds of the profession, and Reform there must be. If the Colleges remain, there are things that probably will not—self-election—irresponsible application of funds—heterogeneous and clashing regulations—local privileges jostling with each other—apprenticeships. These are contrary to the spirit of the times, and neither by force nor art can they very long endure. If the Colleges survive, which, with proper alterations, we believe they may, it becomes a question, whether there should not be some general presiding body, independent of the individual collegiate corporations, and calculated to ensure their future co-operation and consistency. Difficult as it may be to effect this, it will, perhaps, be more difficult, under existing circumstances, to effect any thing beyond this, and, indeed, the Bills of Mr. Hawes and Mr. Warburton seem a practical confession of that difficulty. It is hard to suppose that with those gentlemen's political opinions, they would not have attempted more, if more had appeared to be attainable.

The complicated registrations with which we are threatened would saddle us with expense and harassment, and would expose us to the chicaneries of law. King Log would shortly be King Stork, and our actual rather speculative and political evils would become direct, personal, and social ones. What we want is a well-considered extension of our own, old fashioned, English custom of self-government. What is attempted to be put upon us is a tertium quid between it and centralization. The Government are to have a thumb in the pie—and the lay-people (what are they to us?) are to have another—and we are to put our fingers in;—and a pretty pie it would be! We believe we are uttering the voice of the profession, when we assure the propagandists of this modern political school—something between French and English, with the bad of both—that we do not want them; can do better without them; and their notion of reform is not our's. We would recommend those who are smitten with the centralization passion to read M. de Tocqueville's book. A Frenchman, a profound and original thinker, has utterly sealed its condemnation.

Report says that the Medical Corporations are on the alert, and that meetings have been held with the view of adopting precautionary measures. For our own parts, we would hope that the liberal and enlightened members on the several councils will see the propriety, nay, the necessity of conceding what the state of society and opinion unequivocally demand, and what it must be impossible for any lengthened period to retain. To concede with dignity and promptitude will be to concede with safety; and will tend to prevent that lamentable confusion which will, otherwise, infallibly ensue. We shall be spared such crude and clumsy legislation as Mr. Hawes and Mr. Warburton threaten to inflict upon us.

